# Optimizing nurse staffing in thoracic surgery: the imperative of Enhanced Recovery Programs—a statement of the European Society of Thoracic Surgeons Nursing & Allied Health Professionals Working Group

**DOI:** 10.1093/ejcts/ezae402

**Published:** 2024-11-22

**Authors:** Florian Fehlmann, Johnny Moons, Malene Missel

**Affiliations:** Department of Thoracic Surgery, University Hospital Zurich, Zurich, Switzerland; Department of Thoracic Surgery, University Hospital Leuven, UZ Leuven, Leuven, Belgium; Department of Cardiothoracic Surgery, Copenhagen University Hospital, Copenhagen, Denmark

**Keywords:** Enhanced Recovery Programme, Nurse staffing, Enhanced recovery after thoracic surgery

## Abstract

Enhanced Recovery Programs (ERPs) have revolutionized thoracic surgery by reducing hospital stays and fostering quicker patient recoveries through minimally invasive procedures. However, the perception that patients in ERPs are less complex and require fewer nursing resources is misleading. Despite shorter hospital stays, the complexity of postoperative care remains high, with patients often needing vigilant monitoring and timely interventions. This article challenges the assumption of reduced nursing needs in ERPs, arguing that the fast-paced nature of these programmes intensifies the demand for skilled nursing care. The European Society of Thoracic Surgeons (ESTS) Nurses & Allied Health Professionals Working Group emphasizes that nurse staffing levels must be maintained or even increased to ensure quality care in ERPs. Adequate staffing is crucial for supporting not only the technical aspects of care but also the patient’s experience of illness and recovery. Failure to recognize this complexity could compromise patient outcomes, eroding the benefits of ERPs. This paper advocates for a comprehensive approach that balances efficiency with sufficient nursing support to optimize outcomes in thoracic surgery ERPs. It calls for a reassessment of staffing models to meet the evolving demands of these programmes, ensuring that the advantages of shorter recovery times are not undermined by insufficient care.

## INTRODUCTION

Within the care of thoracic surgery patients, Enhanced Recovery Programs (ERPs) have emerged as a valuable approach, significantly altering patient care trajectories [1]. The implementation of ERPs is associated with reduced hospital stays and less invasive procedures, leading to the perception of patients as less complex [2, 3]. However, amidst these positive changes, a critical aspect that demands attention is the need for adequate nurse staffing to navigate the fast-paced and still highly complex postoperative scenarios.

## CHALLENGING THE ASSUMPTION

Contrary to the assumption that patients in ERPs for thoracic surgery show a reduced complexity, the reality underscores a more nuanced narrative. While these patients may experience shorter hospital stays, the intricacies and intensity of postoperative care remain the same [4]. Additionally, the primacy of caring risks being compromised if ERPs are implemented uncritically, leading to a situation where the individual patient’s life situation is not sufficiently considered [5]. The intricacies of thoracic surgery, even in minimally invasive procedures, amplify the necessity for a highly skilled and well-staffed nursing team. The rapid pace needs seamless coordination, with nurses playing a pivotal role in addressing the unique challenges presented by thoracic surgery patients in ERPs [6]. Acknowledging the unabated complexity of care, it becomes evident that sustaining or augmenting nurse-staffing levels is not only prudent but also imperative for ensuring optimal patient outcomes within thoracic surgery and ERPs.

## ADDRESSING THE NURSE STAFFING CONUNDRUM

To understand the critical nature of nurse staffing in the context of ERPs, it is essential to consider the multifaceted challenges presented by thoracic surgery patients. Furthermore, short hospital stays in ERPs require patients to have co-responsibility for their own care, especially in the transition from hospital to home. Seen from this angle, it becomes particularly clear that specialized thoracic surgical nursing does not end at discharge [7]. The European Society of Thoracic Surgeons (ESTS) Nurses & Allied Health Professionals (AHP) working group emphasizes the importance of maintaining, or even increasing, nurse-staffing levels despite the reduced length of stay associated with ERPs. Evidence suggests that low nurse-to-patient ratios are linked to higher in-hospital mortality rates, increased readmissions and reduced cost-effectiveness [8–10].

A study by Stone and Scheib [11] highlighted that patients in ERPs often experience a swift transition from surgery to recovery, demanding vigilant monitoring and timely interventions. The study underscores the need for sufficient nursing staff to meet the demanding requirements of this accelerated patient care model.

## PATIENT OUTCOMES

While ERPs indisputably display their prowess in support of recovery, unlocking their true potential mandates a comprehensive strategy that places a focus on staffing adequacy. The rapid and streamlined nature of ERPs demands a level of efficiency that hinges on a well-supported nursing team as well as a focus on not just the treatment but certainly also the caring.

The assumption that reduced hospital stays translate into diminished nursing needs overlooks the persistent complexity of care. In this context, faster has often become a corollary to better quality of treatment and care; however, the fact is that the condensed timeframe in which patients move through the various stages of recovery within ERPs intensifies the demand for skilled nursing interventions [12]. This oversight could compromise the very essence of quality care that ERPs promise, potentially eroding the advantages gained through expedited recovery. Considering that thoracic surgery patients are often at an elevated risk of frailty and classified as high-risk individuals [13], it is noteworthy that a higher American Society of Anesthesiologists risk classification is a significant predictor of readmission and increased unplanned outpatient visits within ERPs [14]. Therefore, thoracic surgery patients must be recognized as highly complex also within ERP protocols.

To the authors’ knowledge, no specific evidence exists regarding nurse staffing levels within ERPs. However, substantial research demonstrates that for each additional patient added to a nurse’s workload, the 30-day in-hospital mortality rate rises by 7% in European settings [15]. The findings of Aiken *et al.* [15] also indicate that in hospitals where at least 60% of nurses hold a Bachelor’s degree and care for no more than 6 patients at a time, mortality rates are 30% lower compared to hospitals with only 30% of Bachelor-trained nurses. While the additional costs of professionally trained nurses are a non-disputable fact, the increase of morbidity and mortality in connection with reducing nursing workforce outweighs economic considerations by far [10, 15, 16].

In essence, the success of ERPs relies profoundly on the robust foundation of nursing support and strong interprofessional collaboration. Recognizing and rectifying any underestimation of staffing requirements is paramount, ensuring that the benefits of reduced hospital stays are not offset by a compromised quality of care [9, 10, 14, 15]. Only through a meticulous and foresighted approach to nursing staffing can optimized patient outcomes in thoracic surgery be achieved. Nurses should be supported in fulfilling the mandates of good ERP nursing practice to provide a high quality of patient care, rather than feeling that their best practice is being jeopardized by an organizational system underestimating the need for robust nurse staffing.

## CONCLUSION

In conclusion, the integration of ERPs in thoracic surgery heralds a new era of patient care, emphasizing efficiency and improved outcomes. However, it is imperative to recognize that the swifter pace and falsely assumed reduced complexity of ERP patients do not diminish the need for robust nurse staffing. The ESTS Nurses & AHP working group advocates for a holistic understanding of the intricate demands posed by ERPs, urging healthcare systems to re-evaluate staffing assumptions to ensure the highest quality of care for thoracic surgery patients. Striking the right balance between efficiency and staffing adequacy is the key to unlocking the full potential of ERPs in thoracic surgery.

## Data Availability

No new data were generated or analysed in support of this research.
